# Real time monitoring of COVID-19 intervention effectiveness through contact tracing data

**DOI:** 10.1038/s41598-023-35892-0

**Published:** 2023-06-09

**Authors:** Graham C. Gibson, Spencer Woody, Emily James, Minda Weldon, Spencer J. Fox, Lauren Ancel Meyers, Darlene Bhavnani

**Affiliations:** 1 Los Alamos National Laboratory, Los Alamos, USA; 2 Department of Integrative Biology, University of Texas at Austin, Austin, USA; 3 Department of Population Health, Dell Medical School, University of Texas at Austin, Austin, USA; 4 Epidemiology and Disease Surveillance Unit, Austin Public Health, Austin, USA

**Keywords:** Computational models, Predictive medicine, Statistical methods

## Abstract

Communities worldwide have used vaccines and facemasks to mitigate the COVID-19 pandemic. When an individual opts to vaccinate or wear a mask, they may lower their own risk of becoming infected as well as the risk that they pose to others while infected. The first benefit–reducing susceptibility–has been established across multiple studies, while the second–reducing infectivity–is less well understood. Using a new statistical method, we estimate the efficacy of vaccines and facemasks at reducing both types of risks from contact tracing data collected in an urban setting. We find that vaccination reduced the risk of onward transmission by 40.7% [95% CI 25.8–53.2%] during the Delta wave and 31.0% [95% CI 19.4–40.9%] during the Omicron wave and that mask wearing reduced the risk of infection by 64.2% [95% CI 5.8–77.3%] during the Omicron wave. By harnessing commonly-collected contact tracing data, the approach can broadly provide timely and actionable estimates of intervention efficacy against a rapidly evolving pathogen.

## Introduction

Vaccination and mask wearing continue to be two of the most important public health tools for preventing COVID-19 transmission and mortality^1–10^. However, estimates of their effectiveness vary widely across communities and stages of the pandemic^11^. Measuring the impacts of vaccination and mask wearing is particularly important for protecting vulnerable communities, which include nursing homes and congregate settings such as college campuses^12^. Understanding intervention effectiveness of strategies that have minimal impact on global economic activity can better inform policy responses that balance reduction in epidemic burden and economic cost.

Discrepancies in intervention effectiveness estimates may stem from variation between study populations, such as the prevalence of comorbidities, demographic structure, and contact patterns. During the Delta wave, estimates of two-dose mRNA-1273 (Moderna) effectiveness against symptomatic and asymptomatic infection were as high as 86.7%^13–15^ in a hospital setting and as low as 53.1% in nursing home residents^16^. Although both of these studies controlled for the common sources of heterogeneity (age and previous infection), there may have been a higher prevalence of comorbidities in the nursing home population. During the Omicron wave, effectiveness estimates for two doses of either Moderna or Pfizer mRNA vaccines ranged from 70% in a South African hospital setting^17^ to 36.6% across multiple testing facilities in Ontario, Canada^18^. Again, these studies controlled for many of the same confounding variables, including age and comorbidities, however, Buchan et al. were unable to control for previous levels of infection in the population, possibly leading to the discrepancy in findings^18^. These results highlight that SARS-CoV-2 intervention effectiveness varies through time and across communities and that statistical models for estimating effectiveness should consider local epidemiological, demographic and behavioral conditions.

Test-trace-isolate which includes “contact tracing” has been used widely to prevent COVID-19 transmission, particularly during the early months of the pandemic. While such efforts were often impeded by testing delays and resource limitations^19^, they frequently collected valuable data on the vaccination history, mask use history, behavior, contact patterns, previous infection, and infection status of index patients and their contacts^2,9,20^. Such data allow for estimation of the protective effect of mask wearing and vaccination simultaneously, as well as their effect on both infection and onward transmission. We contrast this with traditional “test-negative” designs of vaccine effectiveness, where symptomatic individuals present to health care providers and their test and vaccine status are recorded. In traditional test-negative designs, confounders are assumed to be randomized conditional on receiving a test (with the exception of healthcare seeking behavior)^21^. However, test negative designs cannot jointly estimate the protective effect of interventions against onward transmission and often have difficulty controlling for previous infection. In the context of COVID-19 test negative designs, it is unclear whether presenting at a testing facility sufficiently controls for an individual’s propensity to wear a mask or social distance. Finally, test negative designs can suffer from susceptible depletion bias^22^, which cannot occur in contact tracing based studies since the analysis is conducted conditional on a set of exposures of susceptible individuals.

We analyze data from a SARS-CoV-2 contact tracing program at a large university in the US to estimate the changing impact of vaccines and face masks on transmission, while highlighting community structures that are higher risk. To our knowledge, this is the first study to estimate vaccine and face mask effectiveness in a university community and the first to simultaneously estimate the effectiveness of more than one intervention in any population, however, other studies have leveraged contact tracing data for vaccine effectiveness estimation^2,23^. Our framework is designed to provide real-time and venue-specific estimates–such as transmission risks associated with schools, workplaces, sporting events, and congregate living–while informing targeted interventions and risk communications.

## Methods

### Definition of intervention effectiveness

We define intervention effectiveness ($\mathbb {E}_A$) as

1 $$\begin{aligned} \mathbb {E}_A = 1-\frac{P(Y_{A=1}=1)}{P(Y_{A=0}=1)} \end{aligned}$$

where $Y_{A=1}$ denotes the counterfactual test positivity rate if everyone had received the intervention and $Y_{A=0}$ denotes the counterfactual test positivity rate if no one had received the intervention. Equivalently, we define the effectiveness of an intervention as one minus the causal risk ratio^21,24,25^. Ideally, we would measure true infections rather than those that test positive, but this is unobservable.

We estimate Eq. (1) from the observed data in the presence of confounding by marginalizing over covariates (*W*).

2 $$\begin{aligned} \mathbb {E}_A = 1-\frac{\mathbb {E}_W[P(Y | A=1,W)]}{\mathbb {E}_W[P(Y | A=0,W)]} \end{aligned}$$

We do not bound the expected value of the effectiveness of the intervention between 0 and 1, but rather allow negative values, which would indicate that the intervention is associated with an increase in the probability of a positive test. To establish $\mathbb {E_A}$ as a causal quantity, we must assume no unmeasured confounding after conditioning on observed covariates as well as counterfactual consistency. We verify that each combination of covariates has at least one individual who tested positive and at least one who tested negative.

As we are particularly interested in the effectiveness across time due to immune waning and emerging variants, we also define the conditional intervention effectiveness, conditional on a particular variant (*V*) dominating during a given time period.

3 $$\begin{aligned} \mathbb {E}_{A|V} = 1-\frac{\mathbb {E}_W[P(Y | A=1,V,W)]}{\mathbb {E}_W[P(Y | A=0,V,W)]} \end{aligned}$$

### Estimation of intervention effectiveness using contact tracing data

We begin by formulating a parametric logistic regression model for the probability of a test positive interaction between an index case and a contact. We denote $Y_{i,j}$ as an indicator of a positive contact of the j-th index case with the i-th contact.

4 $$\begin{aligned} Y_{i,j}\sim & {} \text {Bernoulli}(\mu _{i,j}) \nonumber \\ logit(\mu _{i,j})=\;& {} \beta _0 + \beta _1 \cdot \text {contact masked}_{i,j} + \beta _2 \cdot \text {case masked}_{i,j} \nonumber \\ + & {} \beta _3 \cdot \text {contact vaccinated}_{i,j} + \beta _4 \cdot \text {case vaccinated}_{i,j} \nonumber \\&+ \beta _5 \cdot \text {contact masked}_{i,j} \cdot \mathbb {I(\text {Delta})}_{i,j} + \beta _6 \cdot \text {case masked}_{i,j} \cdot \mathbb {I(\text {Delta})}_{i,j}\\&+ \beta _7 \cdot \text {contact vaccinated}_{i,j} \cdot \mathbb {I(\text {Delta})}_{i,j} + \beta _8 \cdot \text {case vaccinated}_{i,j} \cdot \mathbb {I(\text {Delta})}_{i,j} \nonumber \\&+\beta _9 \cdot \text {contact masked}_{i,j} \cdot \mathbb {I(\text {Omicron})}_{i,j} + \beta _{10} \cdot \text {case masked}_{i,j} \cdot \mathbb {I(\text {Omicron})}_{i,j} \nonumber \\&+ \beta _{11} \cdot \text {contact vaccinated}_{i,j} \cdot \mathbb {I(\text {Omicron})}_{i,j} + \beta _{12} \cdot \text {case vaccinated}_{i,j} \cdot \mathbb {I(\text {Omicron})}_{i,j} \nonumber \\&+ \beta _{13} \cdot \text {duration}_{i,j} + \beta _{14} \cdot \text {indoor}_{i,j} + \beta _{15} \cdot \text {physical contact}_{i,j} \nonumber \\&+ \beta _{16} \cdot \text {relationship}_{i,j} + b_{0,j}\nonumber \\ b_{0,j}\sim & {} N(0,\sigma ^2) \nonumber \end{aligned}$$

In Eq. (4), duration is a binary indicator of the duration of the contact (greater or less than 60 minutes), indoor is a binary indicator of whether the interaction occurred indoors or outdoors, physical contact is an indicator of a direct physical contact, and relationship denotes a categorical variable of the relationship (roommate, classmate, or baseline, which included common relationship types such as friends, partners, co-workers, family). We include an interaction term of the effect of vaccination and mask wearing (for both cases and contacts) with an indicator of the variant circulating during three distinct waves, Alpha, Delta and Omicron. When index cases have multiple contacts, the risks associated with those events may be correlated. To account for this correlation, we include a random intercept ($b_{0,j}$) specific to index case j that captures additional transmission risk beyond the explicit covariates.

We tie this model to the estimand defined in Eq. (3) through a Bayesian parametric formulation of the g-computation formula. The g-computation formula is an established method to marginalize over confounding variables when estimating a marginal treatment effect, in this case, one minus the marginal risk ratio^26,27^. As an example, to recover vaccine effectiveness from Eq. (2) during the Omicron wave we target the following functional of the observed data.

5 $$\begin{aligned} \mathbb {E}_{A|\text {Omicron}} = 1-\frac{\mathbb {E}_W[P(Y_{i,j} |\text {contact vaccinated} =1 , \mathbb {I(\text {Omicron})}_{i,j}=1,W)]}{\mathbb {E}_W[P(Y_{i,j} |\text {contact vaccinated} =0 , \mathbb {I(\text {Omicron})}_{i,j}=1,W)]} \end{aligned}$$

Under a parametric logistic outcome regression model we can plug-in the expression for the expected probability in the numerator and denominator of Eq. (5) and marginalize out over the empirical distribution of confounders.

6 $$\begin{aligned} \mathbb {E}_{A|\text {Omicron}} = 1-\frac{\frac{1}{n_{i,j}}\sum _{i,j} \mathrm {logit}^{-1}(\beta _0 + \beta _3 + \beta _7 + \beta _1 \cdot \text {contact masked}_{i,j} + ....)}{\frac{1}{n_{i,j}}\sum _{i,j} \mathrm {logit}^{-1}(\beta _0 + \beta _1 \cdot \text {contact masked}_{i,j} + \cdots )} \end{aligned}$$

Equation (6) can be thought of as the expected value across the empirical distribution of the data if we were to “set” the contact vaccination status and “set” the circulating variant to Omicron. As each $\beta _k$ is a random variable in a Bayesian context, we obtain uncertainty estimates of our target estimand in Eq. (6) through standard MCMC sampling of the posterior. See Appendix 1 for more details.

## Data

### Contact tracing data

Data were generated by the University of Texas at Austin’s (UT) COVID-19 testing and tracing program which functioned under the authority of Austin Public Health. For the purposes of our analysis, we divided the data into three distinct variant waves; the period when the Alpha variant was dominant (02/15/2021–06/15/2021), when the Delta variant was dominant (06/15/2021–12/10/2021) and when the Omicron variant was dominant (12/10/2021–03/04-2022). We defined a period of dominance by variant as the time period in which greater than 95% of cases in the greater Austin area were attributed to a specific variant. UT Austin reported an average of less than 1% test positivity for both the period when the Alpha and Delta variants were dominant and up to an 8.7% test positivity for the period when Omicron was dominant. The university never implemented a vaccine mandate, but did strongly suggest vaccination. There was a mask mandate in place during the period when Alpha was dominant as well as a hybrid strategy for both in-person and remote classes. However, by the arrival of the Delta variant, the university had lifted the mask mandate and resumed in-person classes with limited remote options. Student, Staff and Faculty cases were reported to UT Contact Tracing following a positive rapid antigen or nucleic acid amplification test conducted between 02/15/2021 and 03/04/2022 (Table 1). Case reports were received from campus testing programs and via self-report. In collaboration with Austin Public Health, UT Contact tracing identified additional UT Student, Staff, and Faculty cases who were interviewed by Austin Public Health. Contact tracers interviewed cases by phone about their symptoms, history of vaccination, and contacts exposed during their infectious period. In 2021, the infectious period was estimated to begin 2 days prior to, and end 10 days after, symptom onset (or, positive test result if asymptomatic) . Beginning on January 1st, 2022, and during the Omicron wave, the infectious period was assumed to end 5 days after symptom onset^28^. Detailed information about the nature of each exposure was collected from the case, including the duration of exposure, context (indoor vs. outdoor), nature of contact (physical vs. non-physical), relationship with the contact (e.g. roommate, classmate, or baseline, which included common relationship types such as friends, partners, co-workers, family) and whether masks were worn by both the case and contact. Contacts were notified of their exposure by phone, and interviewed about their symptoms, history of SARS-CoV-2 infection and vaccination. Contacts with no history of recent infection (within three months of the exposure) were encouraged to seek a COVID-19 test twice, between 3 and 5 days following the exposure to catch new infections early and between 5 and 7 days to align with national guidelines, regardless of vaccination status. Negative test results were acquired through campus testing programs and through self-report. Data was stored in UT’s contact tracing database.

**Table 1:** Characteristics of case-contact interactions by contact test status collected through COVID-19 Contact Tracing at the University of Texas at Austin during the study period (02/15/2021–03/04/2022).

	Negative (*N* = 2612)	Positive (*N* = 511)	Test result unavailable (*N* = 659)	Total (*N* = 3782)
Contact Vaccination Status				
Boosted	109 (4.2%)	38 (7.4%)	25 (3.8%)	172 (4.5%)
Unvaccinated	888 (34.0%)	230 (45.0%)	353 (53.6%)	1471 (38.9%)
Vaccinated	1615 (61.8%)	243 (47.6%)	281 (42.6%)	2139 (56.6%)
Case Vaccination Status				
Boosted	148 (5.7%)	26 (5.1%)	31 (4.7%)	205 (5.4%)
Unkown	86 (3.3%)	94 (18.4%)	10 (1.5%)	190 (5.0%)
Unvaccinated	807 (30.9%)	198 (38.7%)	236 (35.8%)	1241 (32.8%)
Vaccinated	1571 (60.1%)	193 (37.8%)	382 (58.0%)	2146 (56.7%)
Contact Mask Use				
Masked	346 (13.2%)	29 (5.7%)	79 (12.0%)	454 (12.0%)
Unknown	61 (2.3%)	7 (1.4%)	29 (4.4%)	97 (2.6%)
Unmasked	2205 (84.4%)	475 (93.0%)	551 (83.6%)	3231 (85.4%)
Case Mask Use				
Masked	499 (19.1%)	37 (7.2%)	108 (16.4%)	644 (17.0%)
Unkown	61 (2.3%)	7 (1.4%)	29 (4.4%)	97 (2.6%)
Unmasked	2052 (78.6%)	467 (91.4%)	522 (79.2%)	3041 (80.4%)
Exposure Context Location				
Indoor	2525 (96.7%)	472 (92.4%)	639 (97.0%)	3636 (96.1%)
Outdoor	87 (3.3%)	39 (7.6%)	20 (3.0%)	146 (3.9%)
Duration				
15-30 minutes	124 (4.7%)	11 (2.2%)	39 (5.9%)	174 (4.6%)
30-60 minutes	342 (13.1%)	37 (7.2%)	89 (13.5%)	468 (12.4%)
Greater than 60 minutes	1994 (76.3%)	443 (86.7%)	477 (72.4%)	2914 (77.0%)
Less than 15 minutes	59 (2.3%)	8 (1.6%)	19 (2.9%)	86 (2.3%)
Unknown	93 (3.6%)	12 (2.3%)	35 (5.3%)	140 (3.7%)
Days Since Vaccination				
Mean (SD)	169 (68.8)	195 (73.5)	153 (82.5)	170 (72.1)
Median [Min, Max]	169 [1.00, 547]	202 [10.0, 348]	153 [3.00, 415]	170 [1.00, 547]
Missing	857 (32.8%)	227 (44.4%)	342 (51.9%)	1426 (37.7%)
Relationship				
Classmate	708 (27.1%)	16 (3.1%)	117 (17.8%)	841 (22.2%)
Coworker	7 (0.3%)	0 (0%)	1 (0.2%)	8 (0.2%)
Friend	551 (21.1%)	183 (35.8%)	84 (12.7%)	818 (21.6%)
Other	655 (25.1%)	149 (29.2%)	344 (52.2%)	1148 (30.4%)
Partner	100 (3.8%)	39 (7.6%)	45 (6.8%)	184 (4.9%)
Roommate	548 (21.0%)	101 (19.8%)	57 (8.6%)	706 (18.7%)
Teammate	43 (1.6%)	23 (4.5%)	11 (1.7%)	77 (2.0%)
Physical Contact				
Direct non-physical contact (within 6 feet)	1854 (71.0%)	320 (62.6%)	384 (58.3%)	2558 (67.6%)
Direct physical contact	255 (9.8%)	102 (20.0%)	137 (20.8%)	494 (13.1%)
Indirect contact (shared spaces)	430 (16.5%)	80 (15.7%)	105 (15.9%)	615 (16.3%)
Unknown	73 (2.8%)	9 (1.8%)	33 (5.0%)	115 (3.0%)
Contact Tracing Response				
Notified and Screened	2224 (85.1%)	84 (16.4%)	596 (90.4%)	2904 (76.8%)
Unable to make contact	388 (14.9%)	427 (83.6%)	63 (9.6%)	878 (23.2%)

**Figure 1:**
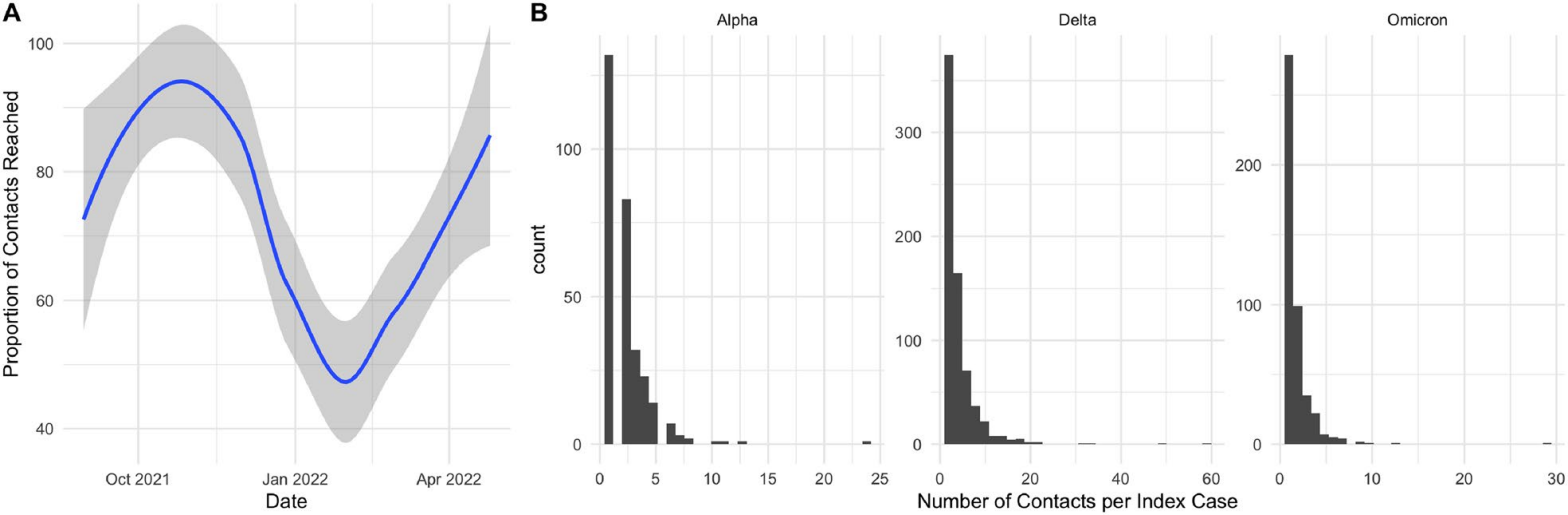
(**A**): Proportion of contacts successfully investigated across time. There was a significant drop in the contact tracers ability to investigate contacts of index cases during the Omicron surge due to the magnitude of the case load. (**B**): Distribution of the number of contacts by index case, conditional on an index case having at least one contact, across the time periods when Alpha (02/15/2021–06/15/2021), Delta (06/26/2021–12/10/2021), and Omicron (12/10/2021–03/04/2022) were dominant.

Cases were included in the analysis if they were: (1) successfully investigated at UT and were willing to share their contacts, (2) had at least one contact who tested at least once 3 to 14 days after the exposure and, (3) had a known vaccination status at the time of exposure. Contacts were included if they had received at least one phone call attempt from contact tracing and if the contact’s vaccination status was known. Test results for each contact were appended to the index case. As we were unable to observe every contact of each index case, and this missingness may be associated with both vaccination status and test positivity, there may be bias in the resulting estimates of vaccine effectiveness. Missingness was particularly pronounced during the period when Omicron was dominant due to an overwhelming case load for contact tracers (Fig. 1A). We explore this bias in detail “Contact tracing data” in section. Most index cases had less than 5 contacts and the distributions of the number of contacts for each of the variant waves appeared roughly power law (Fig. 1B). Any contact that reported a positive test result 3 to 14 days after exposure was considered a test-positive contact. Contacts with only negative results during this period were considered test-negative contacts. Contacts with missing test results were included in an analysis of non-response. Some UT-affiliated contacts who were unable to be notified by contact tracers at UT still tested at a campus testing site and their results were entered into the contact tracing database. Case and contact vaccination status was assessed through two primary routes. First, interviews were conducted over the phone to assess vaccination status at the time of exposure for both cases and contacts. However, when contact tracers were unable to notify a contact and obtain a vaccination status, university vaccination records were used. Cases and contacts were considered fully vaccinated if the exposure occurred at least 14 days after the second dose of an FDA-approved mRNA vaccine. Johnson and Johnson and AstraZeneca vaccinated individuals were dropped from the analysis. Cases and contacts who were unvaccinated, partially vaccinated, or had an exposure occurring in the 14-day period following the final dose of an FDA-approved mRNA vaccine were considered unvaccinated. The sample size was not sufficient to estimate natural waning immunity or booster effectiveness within a period dominated by a particular variant. This study was determined to be not human subjects research by UT’s Institutional Review Board.

### Missing data

A test result was missing for 10.3% of contacts. Unlike traditional test-negative designs, missingness in contact tracing test-negative designs may be informative. If a contact engages in COVID-19 risk taking behavior, they may be more likely to become infected, more likely to be unvaccinated and more likely to be missing a test result. We do observe a lower rate of vaccination among those missing a test (Table 1). Given the integration of testing and tracing data at UT, we were still able to observe individuals who tested at UT regardless of whether the individual was successfully notified by a contact tracer. However, we were unable to observe their test status if they tested off campus or did not test at all. We were also able to monitor the proportion of contacts who were successfully investigated by contact tracers (Fig. 1A). The proportion fell drastically during the Omicron wave, when contact tracers became overwhelmed by the number of cases. If unvaccinated individuals who were likely to test positive due to risk taking behaviors were more likely to *not* respond to contact tracers then this can significantly bias the estimate of vaccine effectiveness downward. In particular, when vaccine effectiveness is low (13%), the bias can reach -100%, indicating that vaccination dramatically increases the odds of testing positive. By monitoring the proportion of contacts successfully notified, we can identify time periods where estimates may become unreliable, such as the Omicron surge. Further details, including specific simulation studies of the bias, are supplied in Appendix A2.

### Ethical approval

All data collection efforts were carried out in accordance with the University of Texas at Austin guidelines and approved by the University of Texas at Austin internal review board (STUDY00000438-MOD02). Informed consent was obtained from all subjects and/or their legal guardians.

## Results

There were 3782 case-contact pairs available for analysis. For the period when the Alpha variant was dominant, there were 300 cases and 593 contacts. For the period when the Delta variant was dominant, there were 703 cases and 2433 contacts. For the period when the Omicron variant was dominant, there were 456 cases and 756 contacts. The distribution of the number of contacts by case is show in Appendix A2. 57% of cases and 56% of contacts were female (Table 1). The median age for index cases and contacts were 21 years (range = 17–48 years) and 20 years (range = 14–40 years), respectively. Over half of all index cases and contacts were vaccinated (56.7% and 56.6%, respectively). The majority of cases and contacts were unmasked at the time of exposure (80.4% and 85.4%, respectively). Exposures typically lasted over 60 minutes (82.2%) and were indoors (96.7%). The average number of days since receiving two doses was 169 for contacts, with 95% having been vaccinated between 50 and 250 days prior to exposure. Overall, 76.8% contacts were successfully notified of their exposure to the index case and 82.5% of contacts tested following their exposure regardless of notification. In order to account for missing test results among contacts, four different scenarios were considered: (1) the contact was notified of the exposure (i.e., answered the contact tracer’s call) and their test result was captured (61.0%); (2) the contact was notified of the exposure but did not get tested (15.7%); (3) the contact was not successfully notified but still tested (21.3%); and (4) the contact was not successfully notified and no test result was available (2%). Test positivity was 3.6% and 52.3% among contacts who were or were not reached, respectively.

**Figure 2:**
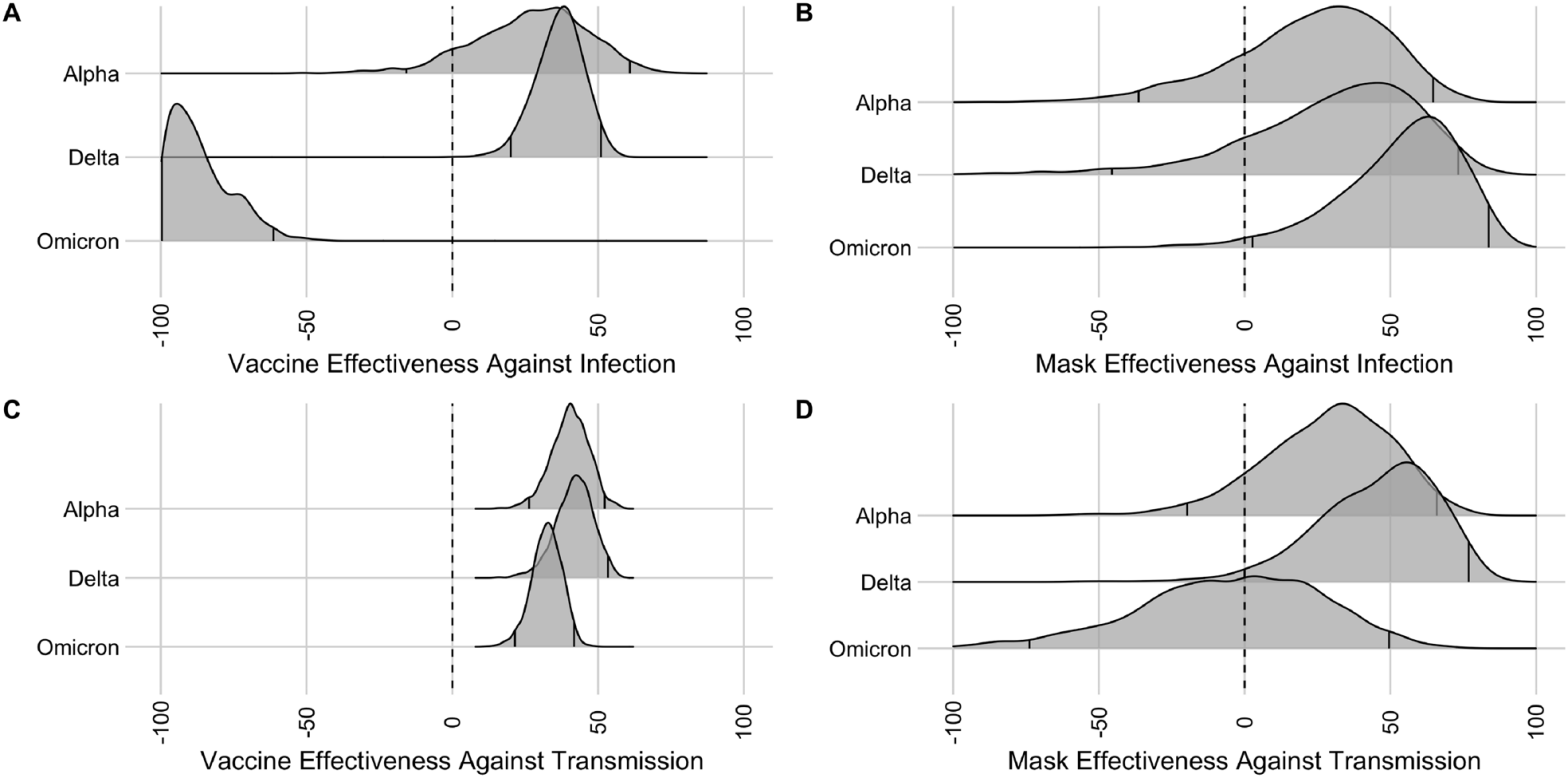
Estimates of intervention effectiveness (in percent) against infection of the contact with 95% credible intervals (vertical black marks). (**A**): Posterior density of vaccine effectiveness against infection (contact vaccination status). The negative estimate for the time period when Omicron was dominant may stem from excess behavioral risks in the vaccinated group. (**B**): Posterior density of mask effectiveness against infection (contact masking status). (**C**): Posterior density of vaccine effectiveness against onward transmission (index vaccination status). (**D**): Posterior density of mask effectiveness against onward transmission (index masking status).

We estimated vaccine effectiveness against infection in the UT community during three different periods of the pandemic (Fig. 2A). When the Alpha variant was dominant (02/15/2021–06/15/2021), vaccination of a contact reduced their risk of infection by 25.1% [95% CI − 16.0–62.0%] based on 493 index case contact pairs (Fig. 2A). When the Delta variant was dominant (06/15/2021–12/10/2021), the estimate was 36.8% [95% CI 20.8–51.3% ] based on 1,885 index case contact pairs. Finally, when the Omicron variant was dominant (12/10/2021–03/04/2022), we found no protective effect, with an estimated effectiveness of -107.0% [95% CI − 159.2 to − 64.8%] based on 294 index case contact pairs. Turning to the vaccination status of the index case, vaccine effectiveness against onward transmission was estimated to be 38.7% [95% CI 23.9–51.1%] during the alpha wave, 40.7% [95% CI 25.8–53.2%] during the delta wave, and 31.0% [95% CI 19.4–40.9%] during the period when Omicron was dominant (Fig. 2C).

Mask wearing by both the contact and the index case was not significantly protective during the Alpha or Delta variant waves (Alpha: 22.7% [95% CI − 36.2–63.9%], Delta: 20.4% [95% CI − 61.8–71.4%] ) (Fig. 2B). However, during the time period when Omicron was dominant mask effectiveness was estimated to be 64.2% [95% CI 5.8–77.3%]. Mask wearing by the index case alone, while not significant, did have point estimates in the direction of a protective effect (Alpha: 26.7% [95% CI − 23.8–63.3%], Delta: 43.4% [95% CI − 8.4–76.9%], Omicron: − 8.0 % [95% CI − 74.2–47.8%]) (Fig. 2D).

**Figure 3:**
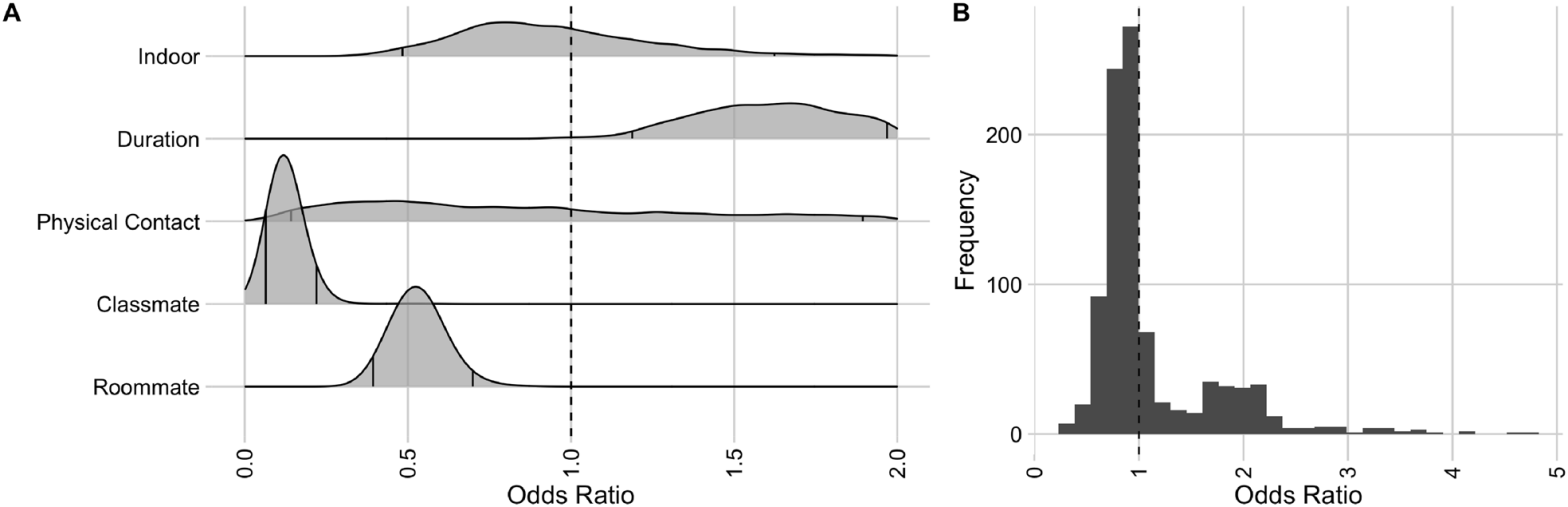
Risk factors for SARS-CoV-2 transmission on a university campus. (**A**): Posterior densities or potentially confounding variables across the study period (02/15/2021 through 03/04/2022), including the relationship between the index case and the contact (classmate, roommate, or baseline, which included common relationship types such as friends, partners, co-workers, family), the location of exposure (indoor vs outdoor), the duration of the interaction (longer or shorter than one hour), and whether there was direct physical contact. The odds ratio indicates the odds for those with the indicator relative to those without the indicator. (**B**): Histogram of the estimated random intercepts of the index cases, representing excess heterogeneity in transmission not explained by the vaccination status of the index case and contact, mask wearing behavior of the index case and contact, relationship between the index case and contact, exposure duration, exposure location, or direct physical contact. Values less than one (greater than one) correspond to index cases who were less (more) infectious than average.

We also estimated the odds ratio of transmission as a function of behavioral and environmental covariates while accounting for vaccination status and masking during the exposure. Classmates (OR = .15 [95% CI .06–.29]) and roommates (OR = .53 [95% CI .39–.70]) (Fig. 3A) were significantly less likely to transmit relative to the baseline, which included common relationship types (friends, partners, co-workers, family). We also find that longer interactions (> 60 minutes) (OR = 1.46 [95% CI 1.13–1.89]) and those involving direct physical contact (RR = 1.37 [95% CI 1.07–1.84]) increase the probability of a test-positive contact relative to shorter and less physical contacts, respectively. The estimated random intercepts by index case exhibit significant variation (Fig. 3B), with some index cases having more than twice the average transmission odds after controlling for covariates. Thus, there are likely other drivers of infection risks beyond vaccination, mask wearing, relationship type, the nature or duration of the exposure, and exposure location.

## Discussion

Contact tracing data from a large urban university in the US revealed a precipitous decline in the effectiveness of vaccines against SARS-CoV-2 infection as the pandemic progressed, consistent with other studies^18,29,30^. Declines likely stem from a combination of immune evasiveness of the variants, natural waning of immunity over the months following vaccination, and unobserved behavioral factors. However, during the Omcron surge, contact tracers became overwhelmed by the volume of cases, and follow up of contacts significantly decreased. This may explain the statistically significant negative estimate of vaccine effectiveness during the Omicron surge and highlights a weakness of the contact-tracing based vaccine effectiveness design. However, by consistently monitoring the rate of successful contact investigations, public health officials can identify periods when estimates may become unreliable. Lyngse et al. similarly found a negative point estimate for vaccine effectiveness against infection during the Omicron wave^31^. The high degree of uncertainty in our estimates of intervention effectiveness stems from small sample sizes, particularly for the periods when Alpha and Omicron were dominant.

We found that vaccines offered a high level of protection against onward transmission during the Alpha, Delta, and Omicron waves. While prior household-based studies have demonstrated this protective effect^32,33^, we believe this is the first indication that vaccines reduce infectivity in a larger community. These estimates provide evidence in support of clear and consistent messaging to promote vaccination, boosting, and vigilant use of masks during dangerous surges in college communities. Contact tracing data allow us to control for environmental and behavioral factors that influence transmission, when estimating the effectiveness of interventions. In our college-aged study population, the relationships between the index case and the contact strongly correlated with infection risk. Individuals who encountered each other through daily college activities (such as classmates and roommates) had a lower odds of testing positive than non-college structured relationships (friends, family, co-workers, etc). These results have counterintuitive implications for university administrators grappling with the costs and benefits of limiting campus activities during surges^34,35^. Limiting in-person classes may be less effective at preventing infection than limiting social gatherings among friends to small “bubbles” or strongly encouraging vaccination, masking and testing to mitigate risks during social encounters.

While large scale studies have confirmed the protective effect of surgical mask wearing^36,37^, in a cluster randomized trial, Abaluck et al. found that mask wearing significantly reduced symptomatic seropositivity by 9%^38^ but the effect decreased for cloth masks relative to surgical masks. This may suggest that our results are driven by the mix of cloth, surgical and other masks used in this college population or data quality issues related to self-reporting of mask use. Larger scale studies are needed to disentangle behavioral from virologic drivers of changes in face mask effectiveness. Our estimates of vaccine and face mask effectiveness in a large university community may be higher than that for a broader US population. College students are more likely to engage in risky behavior in comparison to older adults^39^, and this may be compounded by consistent messaging that COVID-19 is less severe in young adults^39^. College students also tend to have large numbers of close physical contacts per week, given communal living arrangements and classroom structures. They also tend to be young and healthy, with lower prevalence of comorbidities, which may reduce the risks of developing symptoms that would otherwise inhibit social activities while infectious. Even if this larger risk was mitigated by vaccination and face masks, we might still expect lower estimates of intervention effectiveness compared to other populations at lower risk.

While test negative designs often have the advantage of large data sets (high statistical power), consistent monitoring of vaccine effectiveness using contact tracing data can provide complementary real-time estimates that can inform rapid and effective policy responses, particularly when interventions stop working. Unlike test negative studies of vaccine effectiveness, which monitor only symptomatic individuals who present to health care providers, contact tracing data can also catch asymptomatic infections and identify super spreading individuals within a community, potentially helping identify emerging variant threats. However, use of contact tracing data to study intervention effectiveness is prone to three sources of bias that do not occur with the more commonly used test negative design. First, as noted above, missigness can be highly informative and can significantly bias the estimate of vaccine effectiveness (Appendix A2). Second, confounding factors of the particular interaction between the case and contact must be captured retrospectively and controlled for statistically. For example, when studying vaccine effectiveness, one must control for mask wearing since the choice to vaccinate and the choice to wear a face mask may be correlated. Conversely, vaccine status must be considered when estimating masking effectiveness. Finally, contact tracing may involve repeated measurements on a single index case with multiple contacts. These repeated measurements may be correlated due to unmeasured properties of the index case, such as a higher viral load or immunity from prior infections, and must be considered when estimating intervention effects to avoid biased estimates. Other contact tracing based studies^40^ have labeled missing test results as test-negative contacts, potentially biasing the results (Appendix A2). While there is no statistical remedy for missingness not at random, analyses of contact tracing data should include monitoring of contact follow up rates to identify unreliable estimates. We note that data collected by contact tracing programs across the US are highly heterogeneous, ranging from minimal case information^41^ to fully detailing each exposure^42^. Moreover, it is often difficult to link contact tracing data to vaccination records and other relevant health data^42,43^. Increased investment in infectious disease surveillance systems that enhance data capture and integrate healthcare and public health data would facilitate accurate and timely estimation of intervention effectiveness and behavioral risks.

Our study provides a general statistical framework for using commonly collected contact tracing data to track the utility of interventions as the virus continues to evolve and human behaviors shift. The proposed Bayesian g computation model can simultaneously assess multiple interventions, provide probabilistic estimates of their effectiveness at reducing both susceptibility and infectivity, and identify individual- and exposure-related factors that increase risks of transmission. While global contact tracing efforts are winding down, the approach can be applied in real-time as contact tracing is collected in specific populations to provide crucial situational awareness and inform effective mitigation and communication strategies.

### Supplementary Information

Supplementary Information.

## Data Availability

The data that support the findings of this study are available from University of Texas at Austin but restrictions apply to the availability of these data, which were used under license for the current study, and so are not publicly available. Data are however available from the authors upon reasonable request and with permission of the University of Texas at Austin.
