# Supplementary material for: Real time monitoring of COVID-19 intervention effectiveness through contact tracing data
Source: Sci Rep. 2023 Jun 9;13:9371. doi: 10.1038/s41598-023-35892-0 (PMC10250864; doi:10.1038/s41598-023-35892-0)
Supplement: Supplementary file 1 — Supplementary Information. [file 41598_2023_35892_MOESM1_ESM.pdf]

Appendices for "Real Time Monitoring of COVID-19 Intervention Effectiveness through Contact Tracing Data"

April 12, 2023

Contents

1 Appendix A1 **2**

1.1 Causal Risk Ratio Estimation 2

1.2 Simulation Study 3

2 Appendix A2 **5**

2.1 Effect of Network Structure and Missigness on Vaccine Effectiveness Estimation 5

1 Appendix A1

1.1 Causal Risk Ratio Estimation

We begin by outlining the common approach to estimating the causal risk ratio (CRR) in the presence of confounders, which we restate below. Note that intervention effectiveness is one minus the CRR.

$$CRR = \frac{\mathbb{E}_W[P(Y|A=1,W)]}{\mathbb{E}_W[P(Y|A=0,W)]} \tag{1}$$

The CRR of an intervention is most often estimated using a Poisson regression model.

$$\begin{aligned} Y|A,W &\sim \text{Poisson}(\lambda_{a,w}) \\ log(\lambda_{a,w}) &= log(n_a) + \beta_0 + \beta_1 A + \beta_2 W \end{aligned} \tag{2}$$

From Equation 2, we can compute the following expectations,

$$\mathbb{E}_Y(Y|A=1,W) = \lambda_{a=1,w} = n_1 e^{\beta_0+\beta_1+\beta_2 W} \tag{3}$$

$$\mathbb{E}_Y(Y|A=0,W) = \lambda_{a=0,w} = n_0 e^{\beta_0+\beta_2 W} \tag{4}$$

If we take the ratio of Equation 3 and 4 we obtain,

$$\frac{\mathbb{E}_Y(Y|A=1,W)}{\mathbb{E}_Y(Y|A=0,W)} = \frac{n_1 e^{\beta_1}}{n_0} \tag{5}$$

$$\frac{\mathbb{E}_Y(Y|A=1,W)/n_1}{\mathbb{E}_Y(Y|A=0,W)/n_0} = e^{\beta_1} \tag{6}$$

$$\frac{P(Y|A=1,W)}{P(Y|A=0,W)} = e^{\beta_1} \tag{7}$$

Finally, we take the expectation with respect to W of both sides. Note that the expectation is outside the ratio, unlike Equation 1.

$$\mathbb{E}_{\mathbb{W}}[\frac{\mathbb{E}_Y(Y|A=1,W)/n_1}{\mathbb{E}_Y(Y|A=0,W)/n_0}] = \mathbb{E}_{\mathbb{W}}[e^{\beta_1}] \tag{8}$$

However, for a Poisson regression these will be approximately equal, leading to the following estimator for Equation 1.

$$1 - \frac{\mathbb{E}_{\mathbb{W}}[P(Y|A=1,W)]}{\mathbb{E}_{\mathbb{W}}[P(Y|A=0,W)]} \approx 1 - \mathbb{E}_{\mathbb{W}}[e^{\beta_1}] \tag{9}$$

As an alternative to Poisson regression, we can instead directly model the CRR using the parametric g-computation formula. We choose a parametric logistic regression model for $E(Y|A,W)$, which implies,

$$P(Y|A=1,W) = logit^{-1}(\theta \cdot W + \theta_A) \quad (10)$$
$$P(Y|A=0,W) = logit^{-1}(\theta \cdot W) \quad (11)$$

We next take the empirical average over observed covariates values.

$$\mathbb{E}_W[P(Y|A=1,W)] = \sum_{w \in W} logit^{-1}(\theta \cdot w + \theta_A) \quad (12)$$
$$\mathbb{E}_W[P(Y|A=0,W)] = \sum_{w \in W} logit^{-1}(\theta \cdot w) \quad (13)$$

This leads to a natural g-computation based estimator of Equation 1 by taking the ratio of Equation 12 and 13.

$$C\hat{R}R = \frac{\sum_{w \in W} logit^{-1}(\theta \cdot w + \theta_A)}{\sum_{w \in W} logit^{-1}(\theta \cdot w)} \quad (14)$$

However, Equation 14 is only a point estimate of $C\hat{R}R$. To reflect uncertainty in our estimate we adopt a Bayesian approach [1]. We replace parameters (θ, θ_A) with random variables under a specified prior distribution, in our case $N(0, \sigma^2)$. We now use the notation $CRR(\theta, \theta_A)$ to make it clear the causal risk ratio is a function of the random variables θ, θ_A in a Bayesian context.

$$CRR(\theta, \theta_A)|Y, W = \frac{\mathbb{E}_{\mathbb{W}}[P(Y|A=1, W, \theta, \theta_A)]}{\mathbb{E}_{\mathbb{W}}[P(Y|A=0, W, \theta, \theta_A)]} \quad (15)$$

We view Equation 15 as a transformation of the posterior distribution of $(\theta, \theta_A)|Y, A, W$. Therefore, given a sample from the posterior distribution, denoted $(\theta^*, \theta_A^*)|Y, A, W$, we can generate a sample of the posterior over $CRR(\theta, \theta_A)$.

$$CRR^*(\theta, \theta_A)|Y, W = \frac{\mathbb{E}_{\mathbb{W}}[P(Y|A=1, W, \theta^*, \theta_A^*)]}{\mathbb{E}_{\mathbb{W}}[P(Y|A=0, W, \theta^*, \theta_A^*)]} \quad (16)$$

1.2 Simulation Study

To demonstrate the validity of this approach, we perform the following simulation study.

- Sample n individuals.
- For each individual jointly sample their intervention (A) status and their binary cofounder status (W) under a pre-specified correlation ρ and pre-specified marginal probability $p(W=1)$.
- For each individual simulate test status (Y) as follows,

$$P(Y=1|A, W) = logit^{-1}(\beta_0 + \beta_1 \cdot A + \beta_2 \cdot W)$$

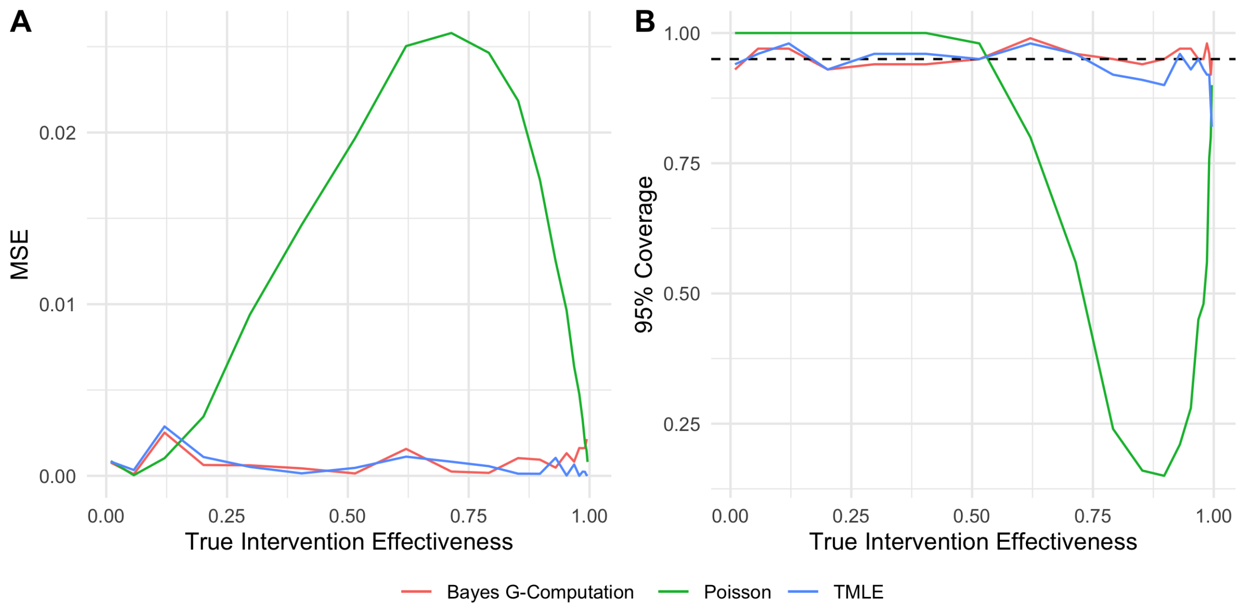

Figure 1: Simulation results comparing Poisson regression estimate of intervention effectiveness to Bayesian parametric g-computation and targeted maximum likelihood (TMLE) [2] estimates of intervention effectiveness evaluated on **A** mean-squared error and **B** 95% coverage.

- As $P(W=1)$ is known, compute the true intervention effectiveness ($\mathbb{E}_A$)

$$\mathbb{E}_A = 1 - \frac{P(Y=1|A=1,W=1)P(W=1) + P(Y=1|A=1,W=0)P(W=0)}{P(Y=1|A=0,W=1)P(W=1) + P(Y=1|A=0,W=0)P(W=0)}$$

- Estimate $\mathbb{E}_A$ using two common approaches (Poisson regression and targeted maximum likelihood) and Bayesian parametric g-computation.

The Bayesian parametric g-computation estimator had lower mean-squared error across almost all values of true intervention effectiveness when compared to a Poisson regression model (Figure 1A). The improvement was particularly pronounced at intervention effectiveness levels above 25% and less than 95%. However, the aboslute value of the MSE remained small ($< .02$) suggesting that Poisson regression models are still relatively unbiased for intervention effectiveness. However, the Bayesian g-computation estimator was much better calibrated than the Poisson regression based estimator (Figure 1B), confirming the result that Poisson regression based estimators of risk ratios from binary data are not well calibrated [3].

When compared to a leading causal estimator, targeted maximum likelihood (TMLE), Bayesian g-computation performs similarly both on MSE and coverage. When all covariates, treatments, and outcomes are binary, the addition of flexible machine learning models used in TMLE may not significantly improve performance.

2 Appendix A2

2.1 Effect of Network Structure and Missigness on Vaccine Effectiveness Estimation

We begin with a description of the simulation study used to quantify the bias introduced only by homophily and heterophily (code available from `https://github.com/gcgibson/ve_simulation`). We then expand the simulation to include preferential missingness of unvaccinated individuals who were more likely to test positive. To simulate an epidemic on a network we perform the following steps. For a given N individuals we perform the following simulation under complete observation.

- Randomly draw a contact network of size N x N from the power law graph generator in the igraph package, assuming power law contact networks are representative of the actual contact network.
- Randomly choose K individuals to be the initially infected index cases OR choose initially infected based on degree distribution.
- Randomly assign vaccination status to each of the K index cases OR choose initially infected vaccination status based on degree distribution.
- For each contact of each index case, randomly assign the contacts vaccination status according to a correlation parameter ρ. This induces either homophily ($\rho > .5$) or heterophily ($\rho < .5$).
- Simulate a single step of an epidemic. Record the successful and attempted transmission pairs i,j along with their vaccination status as contact tracers would do in real time.
- Compute the unadjusted estimator (raw vaccine effectiveness) and our estimator in Equation 3 of the manuscript (adjusted vaccine effectiveness).

We then perform the simulation for a variety of values of the correlation parameter between the index case vaccination status and the contact vaccination status for a given true vaccine effectiveness (Figure 2A). The unadjusted estimator is significantly biased in the negative direction under heterophily and in the positive direction under homophily. However, the adjusted estimator is much less biased. This is because we are explicitly conditioning on the vaccination status of the index case in the adjusted estimator of Equation 3. If unvaccinated individuals preferentially attach to unvaccinated individuals, the regression model will adjust for this.

However, if unvaccinated individuals are more likely to test positive and more likely to be missing there may be significant bias. In order to assess the bias induced in this situation, we augment the simulation study described above and introduce an observation probability on unvaccinated contacts who test positive and are connected to unvaccinated index cases (Figure 2B). In the context of our study, this observation probability may reflect unvaccinated individuals who engage in risk taking behavior and are therefore more

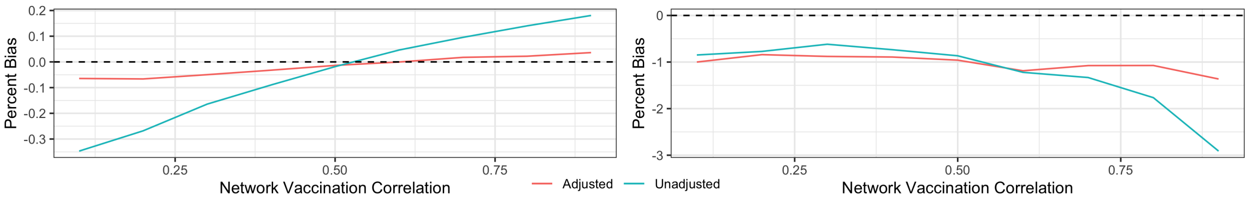

Figure 2: **Left**: Bias in the estimation of vaccine effectiveness under various levels of correlation between index case and contact vaccination status under a powerlaw contact network. **Right**: Bias in the estimation of vaccine effectiveness under various levels of correlation between index case and contact vaccination status under a powerlaw contact network as well as missingness of unvaccinated individuals who test positive.

likely to test positive and less likely to respond to contact tracers. When vaccine effectiveness is particularly low (13%) the magnitude of the bias downward significantly increases (Figure 2B), up to over -100%. We believe this to be the source of the bias of the Omicron estimate of vaccine effectiveness as the percent of successful case contact investigations went down drastically during the Omicron period. The inability to successfully investigate case contact pairs preferentially effected unvaccinated contacts who were more likely to test positive. However, public health officials can monitor the rate of successful follow ups to identify periods when the estimate may become unreliable.

References

[1] Fan Li, Peng Ding, and Fabrizia Mealli. Bayesian causal inference: A critical review. *arXiv preprint arXiv:2206.15460*, 2022.

[2] Mark J Van Der Laan and Daniel Rubin. Targeted maximum likelihood learning. *The international journal of biostatistics*, 2(1), 2006.

[3] Guangyong Zou. A modified poisson regression approach to prospective studies with binary data. *American journal of epidemiology*, 159(7):702–706, 2004.
